# Decrypting the chaperone code

**DOI:** 10.1016/j.jbc.2021.100293

**Published:** 2021-02-16

**Authors:** Andrew W. Truman, Dimitra Bourboulia, Mehdi Mollapour

**Affiliations:** 1 Department of Biological Sciences, University of North Carolina at Charlotte, Charlotte, North Carolina, USA; 2 Department of Urology, SUNY Upstate Medical University, Syracuse, New York, USA; 3 Department of Biochemistry and Molecular Biology, SUNY Upstate Medical University, Syracuse, New York, USA; 4 Upstate Cancer Center, SUNY Upstate Medical University, Syracuse, New York, USA

**Keywords:** Post-translational modifications, Hsp90, Hsp70, Chaperone code, Co-chaperones, HSF1, TRAP1, Extracellular chaperones

The ongoing COVID pandemic has disrupted the ability to hold scientific conferences in person. Despite this, over 200 people from across the world attended The First International Symposium on the Chaperone Code, which was held virtually on October 28 to 29, 2020. The meeting highlighted the many ways that posttranslational modifications (PTMs) on molecular chaperones regulate their function to control proteostasis in diverse organisms.

Cells are continuously exposed to a variety of internal and external stressors that induce protein misfolding. To respond and recover from these stresses, cells express a myriad of molecular chaperone and co-chaperone paralogs that aid in folding, refolding, stabilization, activation, and transport of a large proportion of the proteome (“clients”). While chaperones have been extensively studied for over 40 years, the majority of studies have focused on chaperone actions, ATPase cycle regulation, and *in vitro* folding mechanisms. With the exponential improvement in proteomic technologies over the past 10 years, a huge number of PTMs have been uncovered on chaperones including phosphorylation, acetylation, methylation, SUMOylation, and ubiquitination. Despite the identification of these sites, the respective roles and roles of the modifications (collectively known as the Chaperone Code, Fig. 1) are poorly understood. The inaugural meeting on the Chaperone Code brought together experts from diverse fields ranging from chaperone mechanisms to signal transduction to discuss their latest exciting insights on the Chaperone Code. In this report, we highlight several of these findings in this emerging field of chaperone biology.

**Figure 1:**
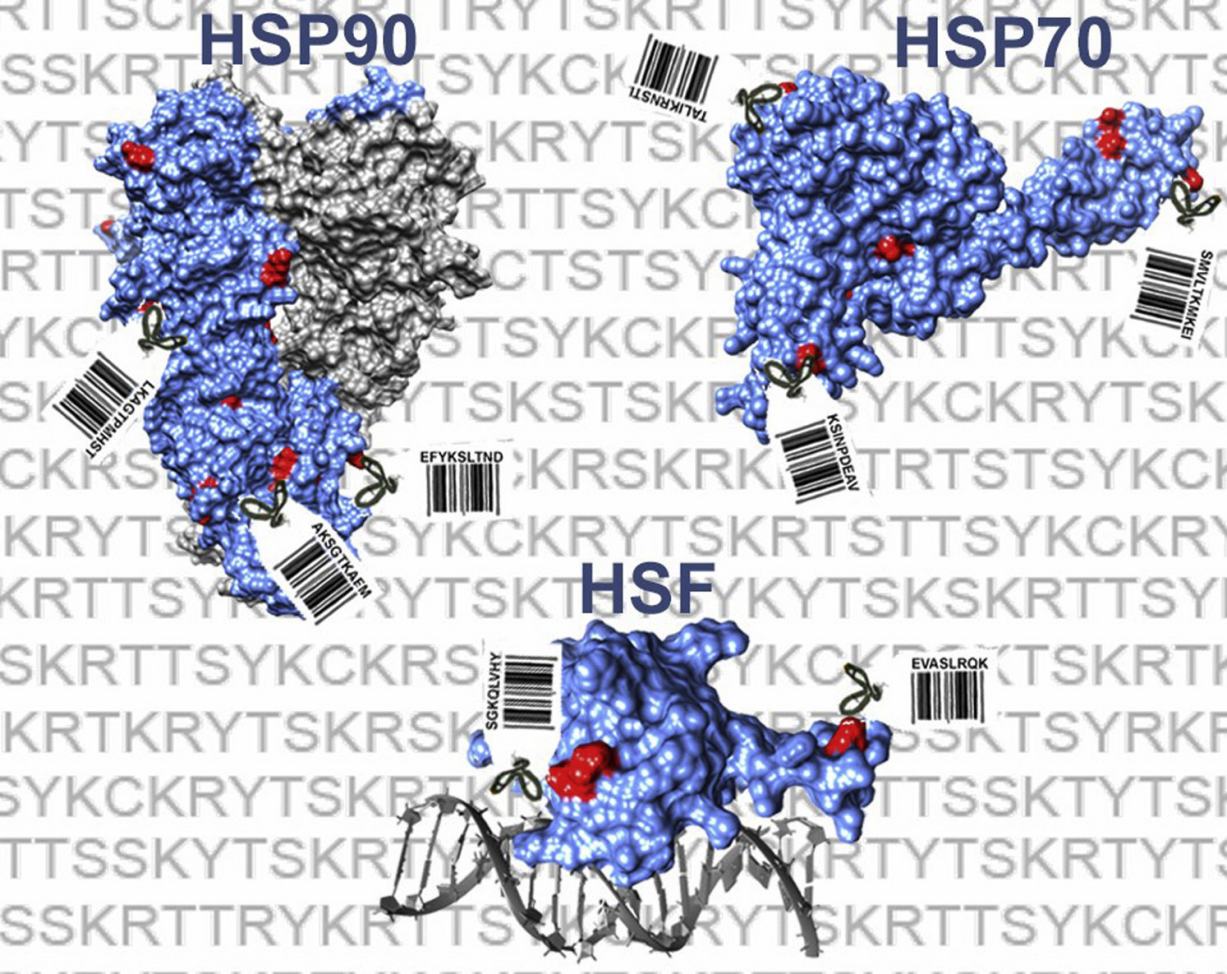
**Schematic illustration of the chaperone code.** The chaperone code comprises all posttranslational modifications on molecular chaperones/co-chaperones, which together create a layer of regulation signified in the illustration by bar codes. Examples discussed in the meeting were Hsp70, Hsp90, and HSF (www.chaperonecode.com).

## Posttranslational modifications of Hsp90

Heat shock protein 90 (Hsp90) is a molecular chaperone involved in folding, stability, and activity of more than 300 proteins also known as clients. Many of these clients are involved in various maladies including cancer, neurodegenerative and infectious diseases (1, 2). Therefore, targeting Hsp90 in preclinical studies and clinical trials in these diseases has been actively pursued (3). Hsp90 chaperone function is linked to its intrinsic ATPase activity of Hsp90. This activity is enhanced by the co-chaperone Aha1 (1).

**Len Neckers** (National Cancer Institute, USA) showed that phosphorylation of a highly conserved Tyr313 in the middle domain of human Hsp90α is important for initial binding of Aha1 (4). Phosphorylation of Tyr313 appears to provide a phosphorylation-sensitive conformational switch that initiates Aha1 C-domain recruitment to the Hsp90 middle (M) domain and consequent stimulation of ATPase activity. This binding pose of the Aha1 C-domain to Hsp90 M-domain, which was unexpected based on previous models, is supported by recently reported orthogonal cryo-EM data (4). Further, since tyrosine phosphorylation of Aha1 also facilitates its binding to human Hsp90 (5), the cooperative action of these PTMs and their impact on protein interaction warrants further investigation.

PTMs of Hsp90 can impact its ATPase activity, suggesting an allosteric regulation mechanism in Hsp90. **Giorgio Colombo** (University of Pavia, Italy) demonstrated the use of computational design in deciphering the chaperone code. Using this approach, it is possible to identify regulatory amino acids that are subject to PTMs and consequently impact the conformational dynamics and ATPase activity of Hsp90 both *in vitro* and *in vivo* (6). This information can ultimately be used to generate small molecule modulators of function or protein–protein interaction inhibitors.

The Hsp90 paralog mitochondrial chaperone tumor necrosis factor receptor-associated protein-1 (TRAP1) is instrumental in metabolic regulation and cell survival. **Andrea Rasola**, (University of Padova, Italy) showed context-dependent PTMs of TRAP1. More specifically, mutation and inactivation of neurofibromin, a Ras GTPase-activating protein, induce an oncogenic metabolic switch *via* mitochondrial ERK-mediated phosphorylation of the molecular chaperone TRAP1 (7).

**Dimitra Bourboulia** (SUNY Upstate Medical University, USA) discussed how secreted kinase signaling could impact extracellular Hsp90 (eHsp90) chaperone function. She showed that the first secretory eHsp90 co-chaperone TIMP2 (8), also an endogenous inhibitor of angiogenesis and regulator of MMP2 activity, is phosphorylated by secreted c-Src tyrosine kinase (9). This modification could define TIMP2 function as an eHsp90 co-chaperone or an MMP inhibitor.

Previous studies have also identified a role for the chaperone code in fungal virulence (10). **Stephanie Diezmann** (University of Bristol, UK) described a new phosphorylation site (S530) on *Candida albicans* Hsp90, targeted by casein kinase 2 (CK2), which results in the inhibition of Hsp90 function and blocking expression of key virulence traits. She showed that the phosphomimetic mutant S530E (but not S530A) abolished *C. albicans* survival at high temperatures, supported a switch to filamentous growth, a morphological change that is important for fungal virulence, and rendered *C. albicans* susceptible to both antifungal (fluconazole) and Hsp90 (radicicol) drugs (11). These studies support the idea that specific Hsp90 PTMs may be exploited as potential antifungal drug targets.

## Posttranslational modifications of Hsp70

The molecular chaperone heat shock protein 70 (Hsp70) is involved in folding, stability, and quality control of proteins (12, 13). Hsp70 is also highly regulated by a range of PTMs (14). **Dalia** **Barsyte-Lovejoy** (University of Toronto, Canada) described how characterization of a novel inhibitor of the arginine methylase PRMT7 led to the discovery that Hsp70 is methylated on R469, a highly conserved amino acid present on the client-binding domain. Intriguingly, this PRMT7-catalyzed methylation can only occur when Hsp70 is in its ATP-bound “open” conformation. R469 methylation appears to be important for a wide variety of chaperone processes that include stress granule response to proteasome inhibition and the general response to heat stress (15).

Lysine methylation comes in several “flavors,” and **Magnus Jakobsson** (Lund University, Sweden) discussed the impacts of mono, di, and trimethylation of Hsp70 K561 promoted by METTL21A. The trimethylated K561 Hsp70 may have the greatest functional relevance, as it is the major form found in both the nucleus and the cytosol (16). Functionally, Hsp70 methylation tunes the interaction of Hsp70 with the disease-associated protein alpha-synuclein (17). Finally, Jakobsson revealed that lysine methylation on Hsp70 in ovarian cancer tumor effusions may be correlated with disease prognosis (18). These fascinating results bolster the growing evidence that multiple mutually exclusive modifications of a single Hsp70 residue can uniquely fine-tune chaperone function and represent biomarkers with clinical utility.

Hsp70 is intimately tied to oxidative stress and metabolic processes in the cell. **Adeleye Afolayan** (Medical College of Wisconsin, USA) demonstrated a novel role of Hsp70 in the mitochondrial import of the superoxide dismutase-2 (SOD2, MnSOD) appropriate to the levels of ROS inside the mitochondria. This process is driven by AKT1-catalyzed phosphorylation of Hsp70 on S631, which alters Hsp70 structure and ability to bind the E3 ubiquitin ligase, CHIP. Several avenues of investigation remain to be explored, including the role of adjacent Hsp70 phosphorylation sites S633 and T636, which are also phosphorylated by AKT1 but do not impact the mitochondrial import of SOD2 (19).

Several talks in the symposium described large-scale structural rearrangements on chaperones in response to PTM addition. **Richard Bayliss** (University of Leeds, UK) described the innovative use of expanded genetic codes in bacteria to produce recombinant Hsp70 phosphorylated on S66. This phosphorylated form was crystallized and revealed that S66 plays a role in Hsp70 interdomain communication. At the cellular level, this Nek6-mediated phosphorylation of Hsp70 is required to maintain a functional mitotic spindle, although the key clients involved are yet to be determined (20).

**Lila Gierasch** (University of Massachusetts Amherst, USA) tested the hypothesis that functionally relevant sites of modifications on Hsp70 chaperone impact its allosteric mechanism. One important site identified was T495 on mammalian Hsp70, a site previously identified in yeast as a functional hotspot as well as being activated in mammalian cells by the *legionella pneumophila* kinase LegK4 as part of its program of infection (21). She is currently exploring the native regulation of this site in yeast and mammalian cells in collaboration with **Andrew Truman** (University of North Carolina at Charlotte, USA).

Cells have evolved to express a multitude of Hsp70 chaperone paralogs (13, 22). Several of these are located at spatially distinct sites. An important example is the ER-resident Hsp70 BiP (also known as Grp78), which is essential for the folding of membrane proteins and those with redox-related modifications such as disulfide bonds. **Seema Mattoo** (Purdue University, USA) described how Huntingtin yeast interacting protein (HYPE; also called FicD) is able to AMPylate the ER-resident Hsp70 BiP at T366 and T518 impacting the BiP ATP cycle. In contrast to many of the other talks demonstrating activation of chaperones through addition of PTMs, her message was that site-specific AMPylation has differential effects on BiP’s ATPase activity and provides a way for cells to stall BiP function in preparation for future stresses (23, 24). Following on from this, **Matthias Truttmann** (University of Michigan, USA) provided evidence that although AMPylation of BiP in *C. elegans* does not alter animal survival, it does reduce Aβ toxicity, an effect that can be mimicked by directly silencing Hsp70 chaperones Hsp1, 3, and 4 simultaneously (25).

## Extending the chaperone code

Although the lion’s share of chaperone code research has focused on the Hsp90 and Hsp70 chaperones, it is now clear that there are many other important regulators of this code. One such example is the heat shock factor (HSF) family of proteins, which regulate the expression of chaperones and co-chaperones under a variety of cellular stresses (26). **Lea Sistonen** (Åbo Akademi University, Turku Bioscience Center, Finland) discussed the extensive stress-induced PTMs of HSFs, exploring their ability to both activate and repress transcription. Using Precision Run-On sequencing (PRO-seq), a technology that maps nascent transcription at a nucleotide’s resolution (27), she showed that HSF1 also binds to highly upregulated enhancers, reprogramming transcription and mRNA expression following heat shock (28).

Although most chaperone PTM studies have focused on phosphorylation, methylation, and acetylation, there are many more that may be important to allow cells to rapidly respond to cellular status. One such PTM is glycosylation, directly regulated through metabolism. Although the interplay between molecular chaperones and metabolism remains poorly defined, it is clear that glycosylation of chaperones and co-chaperones may be key. The dynamic cycling of protein *O*-GlcNAcylation is regulated by the concerted actions of the *O*-GlcNAc transferase (OGT) and the *O*-GlcNAcase (OGA) that add and remove *O*-GlcNAc to serine or threonine amino acids, respectively. **Natasha Zachara** (Johns Hopkins School of Medicine, USA) presented work on decoding the role of glycosylation in stress response. She showed that AMPKα and sequestosome modification by *O*-GlcNac and *O*-GlcNAcylation responds to oxidative stress. Additionally, *O*-GlcNac levels potentiate autophagy in an AMPKα-dependent manner. This is a perfect example of how one PTM such as *O*-GlcNAcylation can be used by cells to sense and respond to stress (29).

**Johannes Buchner** (Technische Universität München, Germany) discussed the consequences of chaperone code failure in the eye lens, loosely interpreting the “code” as the job definition of a chaperone to protect other proteins. Buchner explained that the current model for cataract formation assumes that damaged crystallin proteins assemble into light-scattering aggregates. Chaperones are thought to counteract this by sequestering misfolded crystallin proteins. He showed that in the lens, point mutations in α- (with a chaperone activity), β-, or γ-crystallin proteins are substantially reduced and that the mutant proteins do not accumulate in the water-insoluble fraction. As the mutant lenses show changes in protein composition and the spatial organization of crystallin, this suggests that the imbalance in the lenticular proteome results in changed crystallin interactions as the basis for cataract formation, rather than the aggregation of the crystallin mutants. This work ultimately helps in designing pharmacological treatments to prevent cataract or delay its onset (30).

## Concluding remarks

**Andrew Truman** (University of North Carolina at Charlotte, USA) closed the meeting by describing the history of research uncovering the roles of PTMs in chaperone–client interactions (31). He followed up by summarizing the present state of knowledge on the Chaperone Code, reiterating that despite major advances in the identification of the myriad of PTMs on chaperones, fewer than 5% have been fully characterized. He defined multiple levels of future Chaperone Code research, the first of which is the identification of the stresses and enzymes that regulate specific sites on chaperones, as well as the functional consequences (*in vitro* and *in vivo*) of these modifications. The second level is more complex, determining the interplay between different sites and functional hierarchy involved. In the third level of the Code, evolutionary considerations are taken into account—how do sites of PTM appear on chaperones over time and understanding why different chaperone paralogs have evolved to have different PTMs. At the final level, there was discussion of how all of this information can be integrated into the global concept of the Chaperone Code (14). Given the complexity of the problem faced, the organizers called a united program of collaboration from Chaperone Code researchers and the establishment of monthly Chaperone Code Club Meetings to allow a continuous inclusive dialogue and sharing of ideas to push the field forward.

## Conflict of interest

The authors declare that they have no conflicts of interest with the contents of this article.
